# Genomic abundance is not predictive of tandem repeat localization in grass genomes

**DOI:** 10.1371/journal.pone.0177896

**Published:** 2017-06-01

**Authors:** Paul Bilinski, Yonghua Han, Matthew B. Hufford, Anne Lorant, Pingdong Zhang, Matt C. Estep, Jiming Jiang, Jeffrey Ross-Ibarra

**Affiliations:** 1 Dept. of Plant Sciences, University of California, Davis, Davis, CA, United States of America; 2 School of Life Sciences, Jiangsu Normal University, Xuzhou, China; 3 Dept. of Horticulture, University of Wisconsin-Madison, Madison, WI, United States of America; 4 Department of Ecology, Evolution, and Organismal Biology, Iowa State University, Ames, IA, United States of America; 5 College of Bioscience and Biotechnology, Beijing Forestry University, Beijing, China; 6 Dept. of Biology, Appalachian State University, Boone, NC, United States of America; 7 Genome Center and Center for Population Biology, University of California, Davis, Davis, CA, United States of America; Agriculture and Agri-Food Canada, CANADA

## Abstract

Highly repetitive regions have historically posed a challenge when investigating sequence variation and content. High-throughput sequencing has enabled researchers to use whole-genome shotgun sequencing to estimate the abundance of repetitive sequence, and these methodologies have been recently applied to centromeres. Previous research has investigated variation in centromere repeats across eukaryotes, positing that the highest abundance tandem repeat in a genome is often the centromeric repeat. To test this assumption, we used shotgun sequencing and a bioinformatic pipeline to identify common tandem repeats across a number of grass species. We find that *de novo* assembly and subsequent abundance ranking of repeats can successfully identify tandem repeats with homology to known tandem repeats. Fluorescent *in-situ* hybridization shows that de novo assembly and ranking of repeats from non-model taxa identifies chromosome domains rich in tandem repeats both near pericentromeres and elsewhere in the genome.

## Introduction

Advances in sequencing technology have facilitated development of reference genomes for many non-model organisms, providing a tremendous resource for the field of comparative genomics. Our understanding of the repetitive regions of genomes, however, has lagged behind that of gene-rich regions, mostly because the high identity shared between repeat sequences causes problems with assembly and mapping [1]. Though repetitive DNA is often disregarded as“junk DNA”, research continues to unravel its many functions, spurring a growing interest in a better understanding of the evolutionary history and genomic composition of repeats [2]. Repeat sequence can be broadly classified into two categories: dispersed repeats derived from transposable elements (TEs) and tandemly repeated sequences. TE-derived repeats comprise the majority of many eukaryotic genomes and have been recognized for their potential impacts on phenotype, for example via gene expression [3, 4] or affecting chromatin status [5].

In comparison to the wealth of TE data across organisms, much less is known about the function and evolutionary history of tandem repeats. Tandem repeats are commonly found in the gene-poor regions of the genome such as telomeres and centromeres as well as heterochromatic knobs [6], B chromosomes [7], and sex chromosomes [8]. While tandem repeats generally make up less of the genome than TEs, their abundance varies substantially across phylogenetic groups [9]. In an effort to better understand tandem repeats, researchers have applied both sequencing technologies and molecular biology. Several studies, for example, have paired chromatin immunoprecipitation (ChIP) of centromere proteins with clustering algorithms [10] to identify centromeric repeats [11, 12, 13].

In a recent paper, Melters *et al* [9] conducted *de novo* repeat assembly of published short read sequence data to study the evolution of centromeric tandem repeats across 280 plant and animal species. While tandem repeats do not appear necessary for the formation of centromeres [14], they may serve as placeholders for an epigenetic signal that governs heterochromatin formation [15] or function in repair of double strand breaks [16]. Transcripts from centromere-associated tandem repeats have also been found in the nucleolus of both plant and animal taxa and are thought to be important in protein assembly [17, 18], further suggesting a potential functional role for tandem repeats. Given their likely importance, there is great potential for a bioinformatic approach that takes advantage of published sequence data. One critical assumption of the Melters *et al.* [9] approach, however, is that the most abundant tandem repeat in each genome taxa is the centromere repeat. While comparison to known repeats in several model organisms suggests this assumption works well for animals [9], earlier work suggests that it may not apply broadly to plants. Using a similar pipeline and 454 shotgun reads from *Solanum* taxa, for example, Torres *et al.* [19] identified the most abundant tandem repeats as subtelomeric rather than centromeric.

Here, we test the assumptions of Melters *et al.* [9], applying their pipeline to species within the Andropogoneae tribe of grasses and three outgroups, *Arundinella*, rice, and bamboo, in order to better understand tandem repeat contribution to genomic composition. The Andropogoneae tribe, sometimes referred to as the sorghum tribe, includes both maize and sorghum, two model organisms with well annotated repeats [20, 21]. Many other species in this group are agriculturally and scientifically important, including sugar cane. The presence of well annotated reference genomes allows us to test the accuracy of our method and the Melters *et al.* [9] assumption regarding centromere repeat sequence and its genomic abundance. We examine the genomic composition of highly abundant tandem repeats across these species, determine their homology to known centromere repeats, and perform fluorescent *in-situ* hybridization to test whether novel high-abundance repeats show patterns consistent with known centromere repeats. We show that the common assumption that the highest abundance tandem repeat is centromeric is not supported in these taxa, but that *de novo* tandem repeat assembly can be used to identify entirely novel repeats such as a knob-like repeat in *A. nepalensis* and *U. digitatum*.

## Materials and methods

### Sequencing and genome size measurements

Because previous work has shown that sequencing libraries prepared through identical methods better retain relative composition of repeats [22], rather than use published data we elect to re-sequence all the species used here. Seed was requested from the GRIN database, and accession information is available in Table 1. DNA was isolated from leaf tissue using the DNeasy plant extraction kit (Qiagen) according to the manufacturer’s instructions. Samples were quantified using Qubit (Life Technologies) and 1ug of DNA was fragmented using a bioruptor (Diagenode) with cycles of 30 seconds on, 30 seconds off. DNA fragments were then prepared for Illumina sequencing. First, DNA fragments were repaired with the End-Repair enzyme mix (New England Biolabs). A deoxyadenosine triphosphate was added at each 3’end with the Klenow fragment (New England Biolabs). Illumina Truseq adapters (Affymetrix) were then added with the Quick ligase kit (New England Biolabs). Between each enzymatic step, DNA was washed with sera-mags speed beads (Fisher Scientific). Samples were multiplexed using Illumina compatible adapters with inline barcodes and sequenced in one lane of Miseq (UC Davis Genome Center Sequencing Facility) for 150 paired-end base reads with an insert size of approximately 350 bases. Parsing of reads was performed with in house scripts (All scripts for this and other processes are available at https://github.com/paulbilinski/Github_centrepeat). In short, barcodes were trimmed from the sequence, paired reads were separated so that a single read could be used for assembly, allowing for much faster repetitive contig assembly. Sequence data for each species are available on FigShare (https://dx.doi.org/10.6084/m9.figshare.3494378.v2). Genome sizes were estimated using flow cytometry following [23].

**Table 1:** Counts of reads per sequence library for each taxa. An accession ID of NA indicates a purchase from a local nursery or sample not registered with GRIN. Taxa were selected broadly from across the Andropogoneae tribe, with higher density sampling in the *Tripsacum* genus to study tandem repeat variation within a genus. We used *A. nepalensis*, rice, and bamboo as outgroups to the Andropogoneae. Asterisks indicate genome size estimates published in this study. GS = Genome size.

Genus	Species	Reads	GS (pg/1C)	AccessionID
*Apluda*	*mutica*	746994	1.79*	PI 219568
*Arundinella*	*nepalensis*	662118	2.02[23]	PI 384059
*Hyparrhenia*	*hirta*	861995	1.86*	PI 206889
*Ischaemum*	*rugosum*	920258	0.75*	Kew 0183574
*Oryza*	*sativa*	599567	0.50[24]	NA
*Phyllostachys*	*edulis*	628030	2.1[25]	NA
*Sorghum*	*bicolor*	473944	0.75[24]	PI 564163
*Tripsacum*	*andersonii*	288175	5.8*	MIA 34430
*Tripsacum*	*dactyloides*	391848	3.88[24]	MIA 34597
*Tripsacum*	*floridanum*	743668	3.47*	MIA 34719
*Tripsacum*	*laxum*	723097	3.04*	MIA 34792
*Tripsacum*	*peruvianum*	238983	4.55*	MIA 34501
*Triticum*	*urartu*	435815	4.93[24]	PI 428198
*Urelytrum*	*digitatum*	661535	0.73*	SM3109
*Zea*	*mays*	4422188	2.73[24]	RIMMA0019
*Zea*	*perennis*	5106091	5.28[24]	NA

### Assembly and genomic composition of tandem repeats

To assemble contigs from low coverage sequence, we used MIRA [26] (version 4.0; job = genome,denovo,accurate, parameters = -highlyrepetitive -NW:cnfs = no -NW:mrnl = 200 -HS:mnr = no). We selected to use MIRA over other assemblers due to its relative speed of repetitive sequence assembly without loss of assembly quality. We ran Tandem Repeat Finder [27] (TRF) on assembled contigs, removing any unassembled reads. Previous work has shown that TRF identifies only those contigs that contain tandem repeats [9], and dot plots of the contigs confirmed the presence of tandem repeats (S1 Fig). We utilized only those contigs in all subsequent analyses. Parameters for TRF were Match = 2, Mismatch = 7, Indel = 7, Probability of match = 80, Probability of indel = 10, Min score = 50, and Max period = 2000. Sequence files for all contigs can be found on the project github. To discover the abundance of the tandem repeats identified in our post-TRF analysis contigs, we used Mosaik [28], which stores information about multiply mapping reads (version 1.0; parameters optimized for tandem repetitive elements as in Bilinski *et al* [22]). Low coverage libraries (<0.1X) were mapped against the contigs identified by TRF and contigs were ranked by the number of reads aligned. Previous work has shown that low coverage libraries are sufficient to recover the genomic composition of high abundance repeats [22, 29]. The top ranking contigs above 30bp were extracted, and the number of reads aligning to it was recorded from the assembly ace files. The TRF analysis that identified assembled contigs with tandem repeats also identified the consensus monomer for those tandem repeats. We used the consensus monomer from the top ranking contig to blast (-evalue 1E-1 -outfmt 7 -max_target_seqs 15000 -task blastn; these parameters were used for all BLAST analyses) against all other TRF assemblies and grouped contigs with BLAST homology. The groups of contigs identified by BLAST homology were removed from the contig library and marked as the highest abundance tandem repeat cluster. This process was repeated 4 times to identify the genomic composition of the 4 highest abundance tandem repeat groups; monomer information is available in S1 and S2 Tables. We chose to examine only the top 4 repeats as abundance was often negligible after the 4th repeat. Finally, to estimate the overall abundance of each of these four repeats, we mapped reads against a reference consisting of the most abundant monomer and all polymers with homology to the monomer as determined by BLAST. Mapping against either single monomers or groups of contigs ensured that fragment length bias did not play a large role in overall genomic composition. Contig sequence and length can be found on the project github.

### Fluorescent in-situ hybridization

Repetitive sequences were amplified using the genomic DNA isolated from the targeted species and labeled with digoxigenin-11-dUTP. Hybridization signals were detected with rhodamine-conjugated anti-digoxigenin (Roche Diagnostics USA, Indianapolis, IN). Chromosomes were counterstained with 4‘,6-diamidino-2-phenylindole (DAPI). The following primers were used on the species indicated: *A. nepalensis* (sequence ID 568) Primer F- CCATTCAAGAAATGGTGTCA; *A. nepalensis* Primer R- GCAAGTACGAAAGCCAAAAT; *U. digitatum* (sequence ID 605) Primer F- GCACTGGCCCTGAGAGAAAT; *U. digitatum* Primer R- ACAGGCTTGGGTGGACAAAA; *H. hirta* (sequence ID 520) Primer F- GATCCGAAAGTCGCGAAACG; *H. hirta* Primer R- TTTTTCGCAACGAACGCACA. We were unable to perform FISH on *I. rugosum* due to a lack of living tissue. Primers were designed based on the most abundant tandem repeat contig of the species using the program Primer3 [30]. PCR and FISH was performed using published procedures [31].

## Results

**Fig 1:**
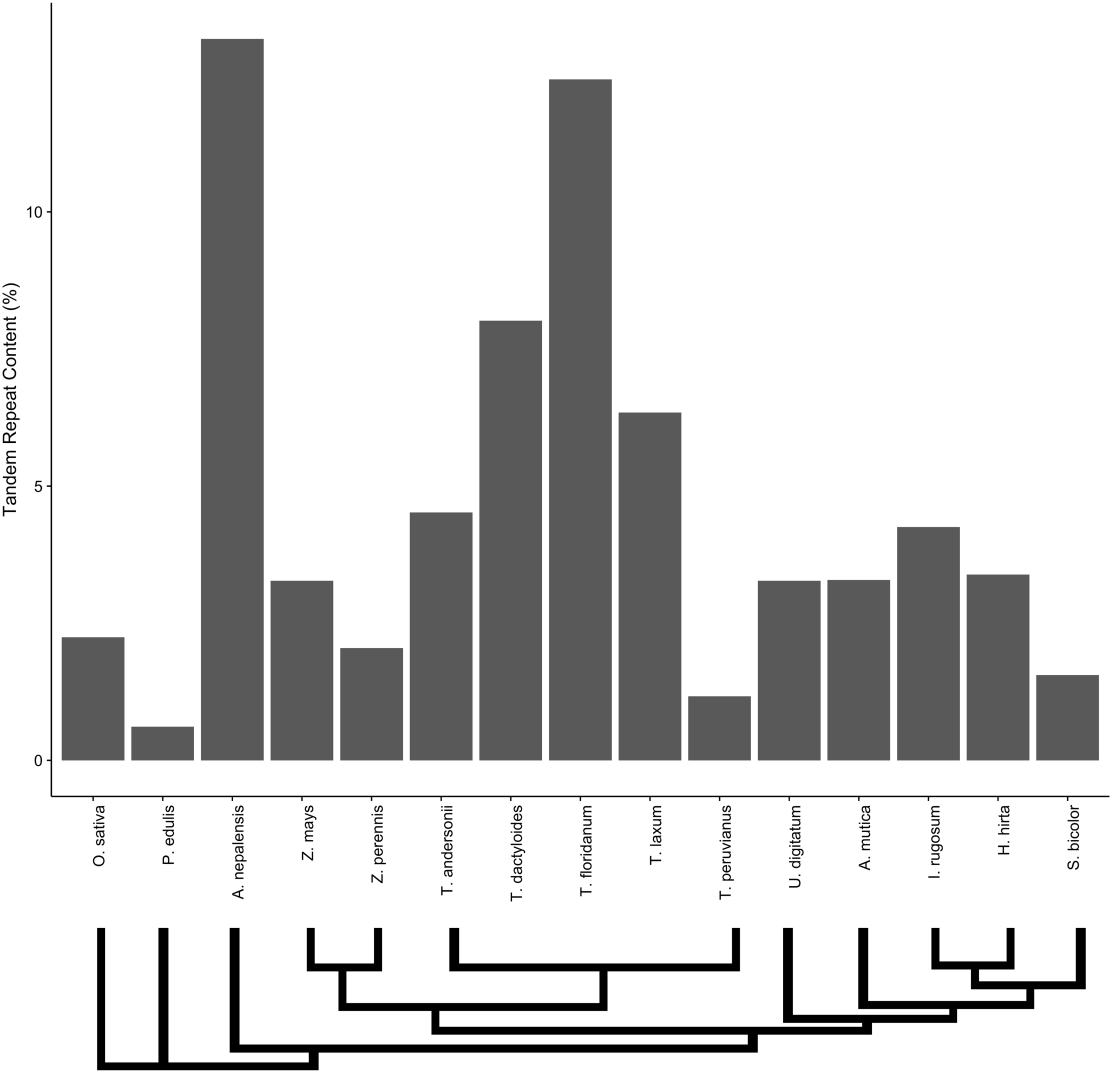
Percentage genomic composition of all tandem repeat contigs in monocot taxa. Values are derived from the proportion of all reads mapping to any tandemly repetitive contig derived from TRF after MIRA assembly. Species are ordered in approximate phylogenetic relationship, with a phylogenetic schematic below the graph.

Assembly of low depth resequencing data produced several thousand contigs in each species from our panel (Fig 1, and Table 1). From these, TRF identified between 300 and 15,000 tandem repeat contigs in each taxon (sequences available on the project github at https://github.com/paulbilinski/Github_centrepeat). The number of tandem repeat contigs varied across taxa based on coverage and overall genomic repetitive content. We then mapped our sequence data against tandem repeat contigs to approximate the abundance of tandem repeats in our panel (Fig 1). Our taxa vary greatly in their total tandem repeat content, ranging from over 13% to under 1%. We see high tandem repeat content across the *Tripsacum* genus and in *A. nepalensis*, though *Tripsacum* taxa show large variation. Based on genome size estimates (see Table 1), the correlation between total tandem repeat content and genome size is poor across all taxa as well as within *Tripsacum* (Pearson correlation; p >0.05).

**Fig 2:**
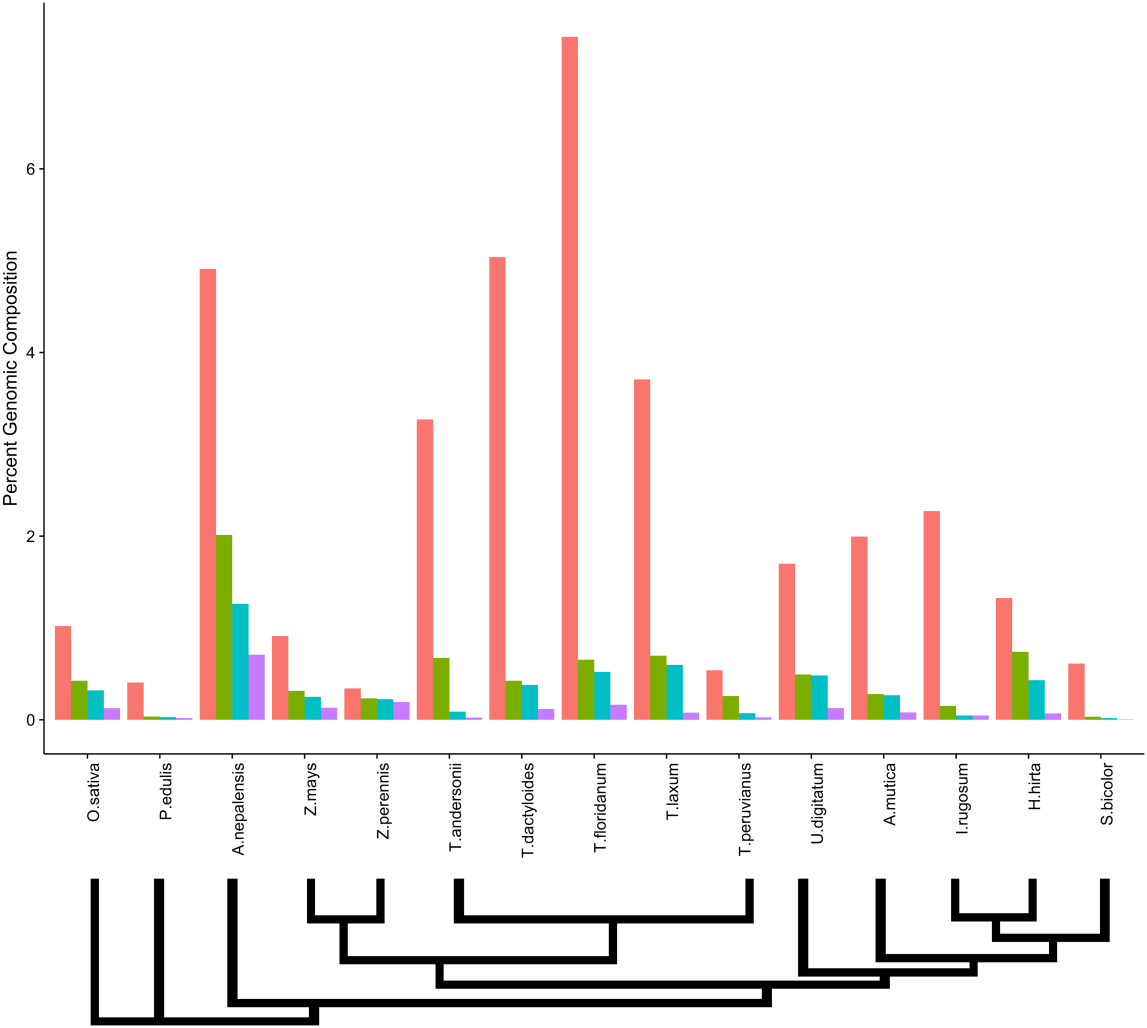
Genomic composition of top 4 tandemly repetitive contigs. The top 4 contigs in each species were defined as not having homology to one another, in order to identify independent repeat motifs. Species are ordered in approximate phylogenetic relationship, with a phylogenetic schematic below the graph. Values were calculated as a percentage of total genomic reads mapping to each tandem repeat family. Tandem repeat families are ordered by their genomic abundance from left to right.

In order to investigate the proportional contribution of the most common tandem repeat classes in each of the analyzed taxa, we ranked the mapping abundance of all contigs containing tandem repeats as identified by TRF. We used the number of reads mapping to the top ranked contig as its abundance, and removed any similar contigs from our rankings using BLAST homology (see methods for parameters). We repeated this for the top four tandem repeats in each genome. Results showed that most taxa had one tandem repeat at much higher abundance (Fig 2). In all taxa except for *A. nepalensis*, only the top contig exceeded 1% of genomic composition. *S. bicolor*, *P. edulis*, *I. rugosum*, and *A. mutica* showed the largest difference between the top ranked contig and the second ranked contig. In the sister genera *Zea* and *Tripsacum*, while the top ranked contig showed immense variation, the second ranked contig had a relatively constant abundance near 0.5%.

**Fig 3:**
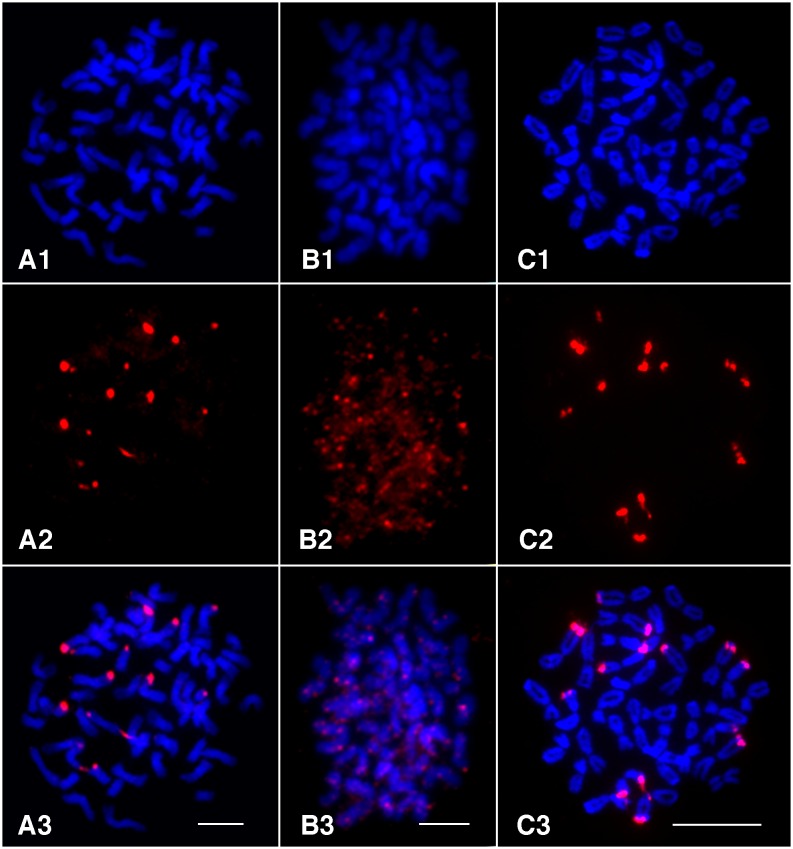
Fluorescent in situ hybridization of the highest abundant tandem repeats in three grasses. (A1-C1) Somatic metaphase chromosomes prepared from *A. nepalensis* (A1), *H. hirta* (B1), and *U. digitatum* (C1), respectively. (A2-C2) FISH signals derived from the three repeats identified in the three species. (A3-C3) Images merged from chromosomes and FISH signals. Scale bar = 10 microns. On all images, knobs are indicated with white arrows.

We wanted to test whether the assumption that the most abundant repeat is centromeric [9] could be applied to these grass taxa with both known and uncharacterized centromere repeats. Among taxa with known centromere repeats, the centromere repeats were found to be the most abundant tandem repeat in both *O. sativa* and *S. bicolor*. The percentages of the genome comprised from each tandem repeat was similar to other studies performed in both Sorghum (1.6–1.9%) [32] and maize(<1%) [21]. In *Zea* and the closely related *Tripsacum* taxa, the centromere repeat was among the four most abundant, but the highest abundance repeat came instead from heterochromatic knobs, as has been noted previously [9, 33]. In *P. edulis*, the most abundant repeat has homology to a repeat region in *Bambusa*, but is not annotated as centromeric. While the centromere repeat was not previously known for the species *A. mutica*, its highest abundance contig shared homology and a common monomer repeat length with the *S. bicolor* centromere repeat. Previous FISH studies have shown that the *Tripsacum* centromere repeat shares homology to maize and is localized to the centromere [34]. Our data show that the ranking of the top two repeats in all *Tripsacum* species studied is the same, while the 3rd and 4th most abundant repeats vary between the species within the genus. The top-ranked contig in *I. rugosum* shared a monomer length identical to the centromere repeat of *Sorghum*, but with no sequence homology. The top ranked contigs from the remaining taxa in our panel bore no similarity to known centromere repeats. *T. urartu* did not have any tandem repeats longer than 30 bp at an appreciable frequency in the genome (see Methods). To test whether the most abundant repeat in these taxa is centromeric, we performed fluorescent *in situ* hybridization on *A. nepalensis*, *H. hirta*, and *U. digitatum* (FISH; Fig 3), expecting spatial clustering of the probe proximal (for metacentric) or distal (for acrocentric) of most if not all chromosomes. FISH from the *de novo* constructed repeat of *H. hirta* is widely dispersed across the genome, a pattern reminiscent of a TE rather than a localized tandem repeat. The tandem repeat from *U. digitatum* showed strong spatial clustering, though clusters were not found on all chromosomes and were associated with chromosome ends as might be expected from a subtelomeric sequence. The regions probed in *U. digitatum* did associate with visible knobs and the monomer repeat length is 184bp, similar to the 180bp knob repeat found in tightly packed heterochromatin in *Zea* (Fig 3). The monomer sequence of *U. digitatum* does not have homology longer than 30bp to any annotated sequence and may be a novel knob variant identifed here. The probed repeat of *A. nepalensis* also showed subtelomeric clustering, and the fact that *A. nepalensis* had the largest proportion of its genome comprised of tandem repeats (Fig 1) is consistent with a knob-like origin for this tandem repeat. In both *A. nepalensis* and *U. digitatum*, FISH signal did not occur at visible primary constriction sites (Fig 3). While the suspected knob repeat sequences in *A. nepalensis* had sequence lengths similar to those in maize (approximately 180bp and 350bp), the sequences share no identity. Our *A. nepalensis* FISH also showed that the tested 180bp probe did not bind to all visible knobs, and we speculate that FISH using the 350bp repeat, which ranks second in abundance, would likely bind to some of the other visible knobs. From these FISH results, we conclude that genomic abundance is not predictive of centromere localization in the Andropogoneae.

## Discussion

Our analyses of *de novo* assembled tandem repeats in grasses provides insight into the utility of this approach for studying the evolution of repetitive sequences. Most importantly, we show that previous assumptions about repeat abundance and location within the centromere do not hold across all taxa. Identification of the most abundant tandem repeat failed to identify centromeric repeats across many taxa, though in some cases it did identify sequences with homology to known centromere repeats. In *Tripsacum* taxa, previous work has shown that the maize tandemly repeated centromere element CentC cross-hybridized to the *Tripsacum* centromeres [34, 35]. As our FISH data show, *de novo* assembly and abundance ranking identified non-centromeric repeats in all taxa whose most abundant repeat did not share homology with a known repeat. Given the inconsistency of abundance as a predictor of centromere localization, we believe the alternative method of chromatin immunoprecipitation [13], despite its higher costs, is likely a more accurate and better method to reliably identify centromere repeats. Also, new sequencing technologies, such as Pacific Biosciences long reads, can also be helpful in studying tandem repeats [22]. As costs decrease, long reads can be used alongside ChIP studies to identify higher order structure in tandem repeats and eventually assemble long tandem repeat arrays.

Though not ideal for centromere repeat identification, *de novo* assembly of tandem repeats can be an efficient, low cost method for characterizing repetitive content in non-model genomes. Our assembly of *A. nepalensis* and *U. digitatum* repeats serve as examples of novel findings that can be made regarding repeat sequences using this approach. *A. nepalensis*, sister to Andropogonae, has two highly abundant tandem repeats that do not share homology to any annotated genetic sequence, but are of similar sequence lengths of 180bp and 350bp as knob repeats in *Zea* and *Tripsacum* and found in knob-like heterochromatin. *U. digitatum* is similar, with the high abundance 184bp repeat associated with visible knobs and lacking homology >30bp to annotated sequence. Like in *Zea*, the *A. nepalensis* 180bp repeat is the highest abundance tandem repeat, and the 350bp tandem repeat is the next highest abundance tandem repeat with a different length (S1 and S2 Tables). While the sequence length of the tandem repeats are similar to those observed in many subtelomeric repeats [19], we speculate that the high genomic abundance of both the *A. nepalensis*, *U. digitatum*, and *Zea* may suggest that these new repeats are also knob-like. Knobs are associated with meiotic drive in maize [36] and suppress recombination locally but increase recombination in the intervening region between themselves and the centromere [37]. Knobs are known in a number of other plant taxa, such as maize, *Tripsacum*, rye [38], and *Arabidopsis thaliana* [39]. That we find no sequence homology between *A. nepalensis* or *U. digitatum* knobs and those in *Zea* suggests we may have identified a novel knob repeat that comprises a disproportionate fraction of the genome comparable only to certain maize and *Tripsacum* taxa, while not sharing homology to any known repeat. Further work will be necessary to identify whether the putative knobs of *A. nepalensis* or *U. digitatum* function similarly to those in maize with regard to recombination and meiotic drive, and analysis of additional taxa may reveal whether the accumulation of knobs near chromosome ends is also a common evolutionary theme [40].

The methods presented here can also be applied to study variation in genomic composition within and between species. Genome size is highly variable across plants and is associated with many important phenotypic traits such as flowering time and seed size [41, 42]. The ability to identify the percentage of the genome composed of specific types of tandem repeats can enable studies that track the components driving genome size variation. For example, identification of genomes with high abundance tandem repeats may lead to a better understanding of selfish genetic elements and how they influence long term evolution. Altogether, the results presented here show how *de novo* assembly can be used to better understand the repetitive fraction of the genome.

## Supporting information

S1 Fig Dot plot of the *A. nepalensis*, *T. laxum*, and *H. hirta* highest abundance contigs against themselves. Lines indicate share sequence identity.
(TIFF)

S1 Table Percentage genomic composition of the top four tandem repeat groups. Species are ordered phylogenetically.
(PDF)

S2 Table Monomer information for taxa studied. (PDF)
