# Supplementary figures and images for: Genomic abundance is not predictive of tandem repeat localization in grass genomes
Source: PLoS One. 2017 Jun 1;12(6):e0177896. doi: 10.1371/journal.pone.0177896 (PMC5453492; doi:10.1371/journal.pone.0177896)

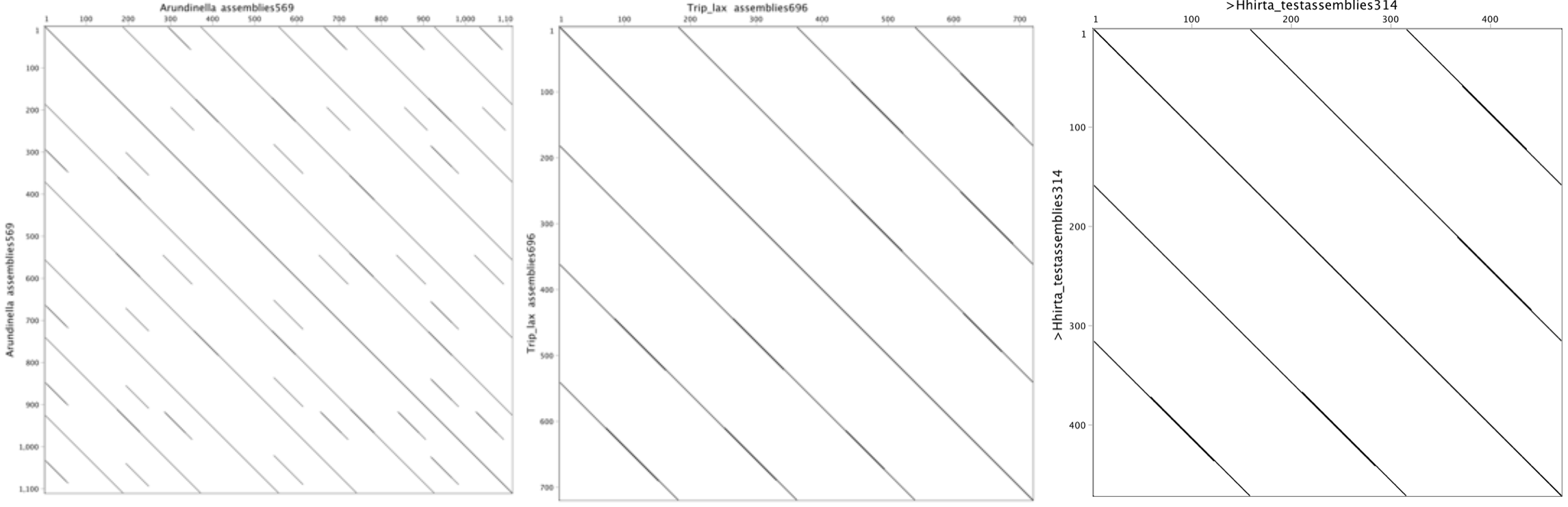

Supplement: S1 Fig — Lines indicate share sequence identity. (TIFF) [file pone.0177896.s001.tiff]
