# Supplementary material for: Genomic abundance is not predictive of tandem repeat localization in grass genomes
Source: PLoS One. 2017 Jun 1;12(6):e0177896. doi: 10.1371/journal.pone.0177896 (PMC5453492; doi:10.1371/journal.pone.0177896)
Supplement: S1 Table — Species are ordered phylogenetically. (PDF) [file pone.0177896.s002.pdf]

S1 Table. Percentage genomic composition of the top four tandem repeat groups. Species are ordered phylogenetically.

Taxa	First	Second	Third	Fourth
O. sativa	1.021	0.425	0.320	0.126
P. edulis	0.405	0.034	0.027	0.018
A. nepalensis	4.911	2.012	1.263	0.708
Z. mays	0.912	0.316	0.251	0.132
Z. perennis	0.341	0.232	0.224	0.194
T. andersonii	3.271	0.674	0.087	0.025
T. dactyloides	5.041	0.425	0.379	0.118
T. floridanum	7.438	0.653	0.521	0.165
T. laxum	3.707	0.698	0.597	0.077
T. peruvianus	0.539	0.259	0.073	0.026
U. digitatum	1.700	0.493	0.484	0.127
A. mutica	1.995	0.280	0.267	0.081
I. rugosum	2.272	0.151	0.046	0.046
H. hirta	1.326	0.741	0.432	0.069
S. bicolor	0.612	0.033	0.017	0.003
