# Supplementary material for: Genomic abundance is not predictive of tandem repeat localization in grass genomes
Source: PLoS One. 2017 Jun 1;12(6):e0177896. doi: 10.1371/journal.pone.0177896 (PMC5453492; doi:10.1371/journal.pone.0177896)
Supplement: S2 Table — (PDF) [file pone.0177896.s003.pdf]

S2 Table. Monomer information for taxa studied.

Taxa	Repeat Rank	Length	ID
O. sativa	1	155	260
O. sativa	2	352	150
O. sativa	3	246	0
O. sativa	4	113	63
P. edulis	1	112	145
P. edulis	2	285	60
P. edulis	3	191	67
P. edulis	4	200	193
A. nepalensis	1	185	568
A. nepalensis	2	185	827
A. nepalensis	3	369	836
A. nepalensis	4	103	1140
Z. mays	1	179	51
Z. mays	2	642	117
Z. mays	3	580	4215
Z. mays	4	80	200
Z. perennis	1	117	350
Z. perennis	2	180	229
Z. perennis	3	630	4959
Z. perennis	4	156	3862
T. andersonii	1	180	75
T. andersonii	2	156	509
T. andersonii	3	248	10
T. andersonii	4	184	383
T. dactyloides	1	180	109
T. dactyloides	2	156	1129
T. dactyloides	3	359	54
T. dactyloides	4	171	13
T. floridanum	1	179	1561
T. floridanum	2	156	90
T. floridanum	3	359	3205
T. floridanum	4	171	128
T. laxum	1	180	694
T. laxum	2	156	255
T. laxum	3	359	66
T. laxum	4	214	54
T. peruvianus	1	180	11
T. peruvianus	2	156	110
T. peruvianus	3	359	33
T. peruvianus	4	203	36
U. digitatum	1	184	752
U. digitatum	2	102	262
U. digitatum	3	190	164
U. digitatum	4	186	842
A. mutica	1	138	123
A. mutica	2	154	287
A. mutica	3	335	17
A. mutica	4	579	905

I. rugosum	1	137	2548
I. rugosum	2	50	3103
I. rugosum	3	522	3313
I. rugosum	4	503	1828
H. hirta	1	157	313
H. hirta	2	185	323
H. hirta	3	183	225
H. hirta	4	183	45
S. bicolor	1	137	58
S. bicolor	2	123	7
S. bicolor	3	98	4
S. bicolor	4	180	17
